# Supplementary material for: Impact of the Area of Residence of Ovarian Cancer Patients on Overall Survival
Source: Cancers (Basel). 2022 Dec 4;14(23):5987. doi: 10.3390/cancers14235987 (PMC9736843; doi:10.3390/cancers14235987)

Table S1. List of sociodemographic variables

Category	Variable
**Income/Poverty**	Median family income ($)
	Median household income ($)
	Percent of persons whose incomes are below the poverty level
	Percent of persons who are below 150 Percent of the poverty level
	Percent of persons who are below 200 Percent of the poverty level
	Percent of families whose incomes are below the poverty level
**Education**	Percent with less than 9th grade
	Percent with less than high school graduate
	Percent with at least a bachelor's degree
**Demography**	Percent of persons under age 18
	Percent of persons ages 65 and over
**Employment**	Percent of persons ages 16 and over who are unemployed
**Housing**	Percent of households with more than one person per room
**Immigration**	Percent of persons who are foreign born
	Percent of households that are linguistically isolated
**Smoking**	Percent of current smorker
	Percent of people who have ever smoked
**Medical follow-up**	Percent of persons with a mammography within 2 years
	Percent of persons with a Pap smear within 3 years
	Percent of people who have ever had a CRC test
	Percent of people who have ever had an endoscopy
	Percent of people who have ever had a FOBT test
**Rural/Urban**	Percent of people living in urban areas
**Mobility**	Percent of persons aged 5 and over in the same house than last year (no migration)
	Percent of persons aged 5 and over moved but in the same county
	Percent of persons aged 5 and over moved from a different county but in the same state
	Percent of persons aged 5 and over moved from a different state in the United States
	Percent of persons aged 5 and over moved from outside the United States

Figure S2. Results of the PCA
Plot A represents the variance explained by each dimension.
Plot B shows the contribution of each sociodemographic variable for the two first dimensions.

A

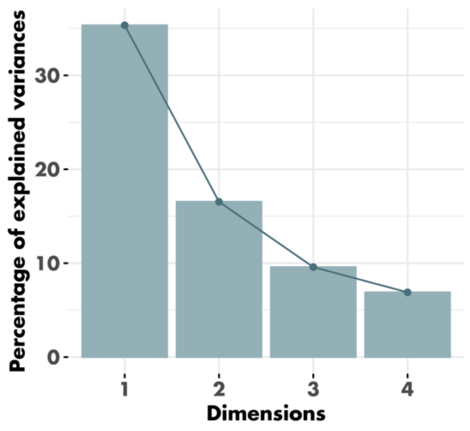

B

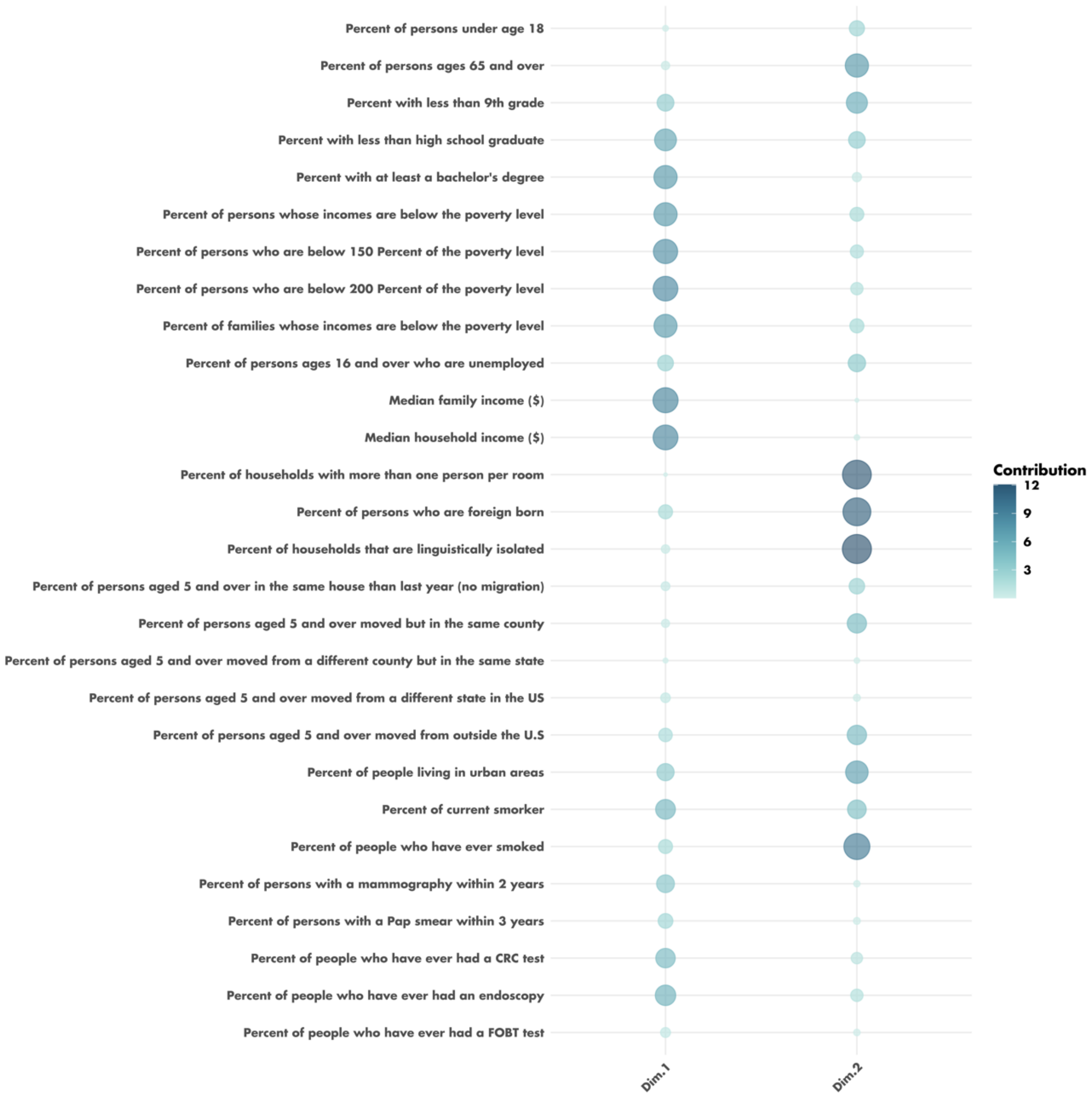

Figure S3. Distribution of the variables related to demography in the four clusters

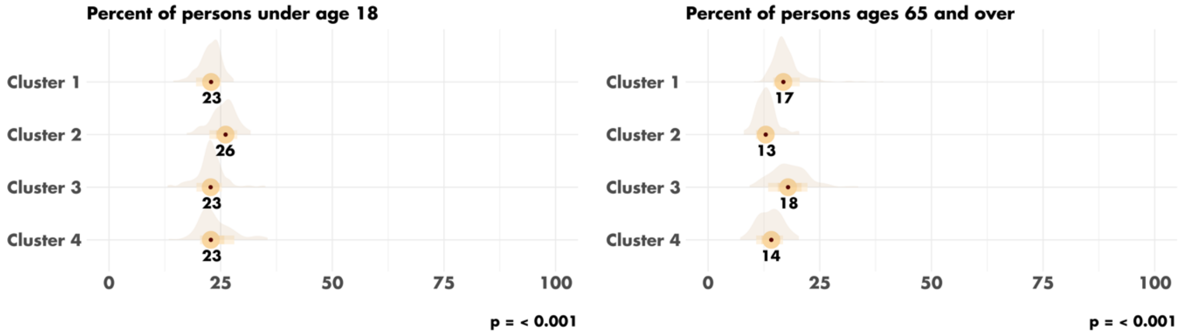

Figure S4. Distribution of the variables related to housing in the four clusters

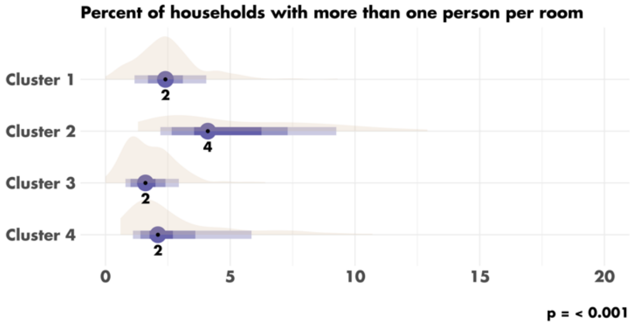

Figure S5. Distribution of the variables related to smoking in the four clusters

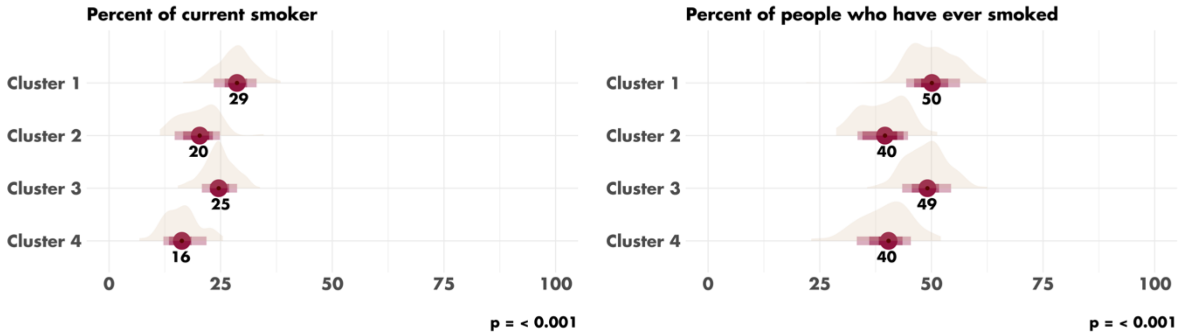

Figure S6. Distribution of the variables related to mobility in the four clusters

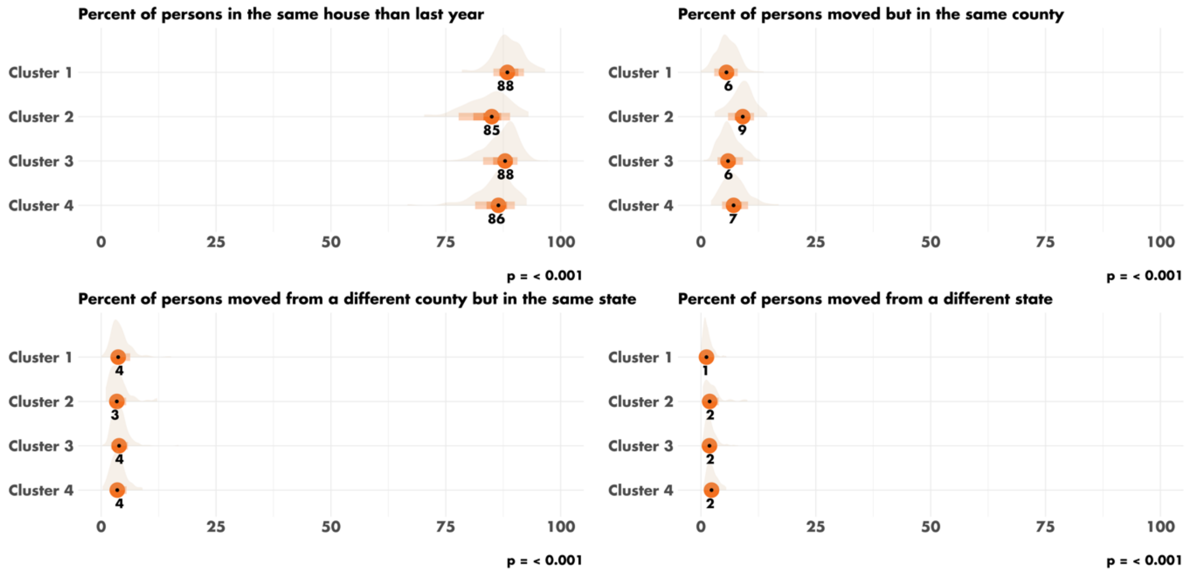

Figure S7. Distribution of metastasis combination by cluster

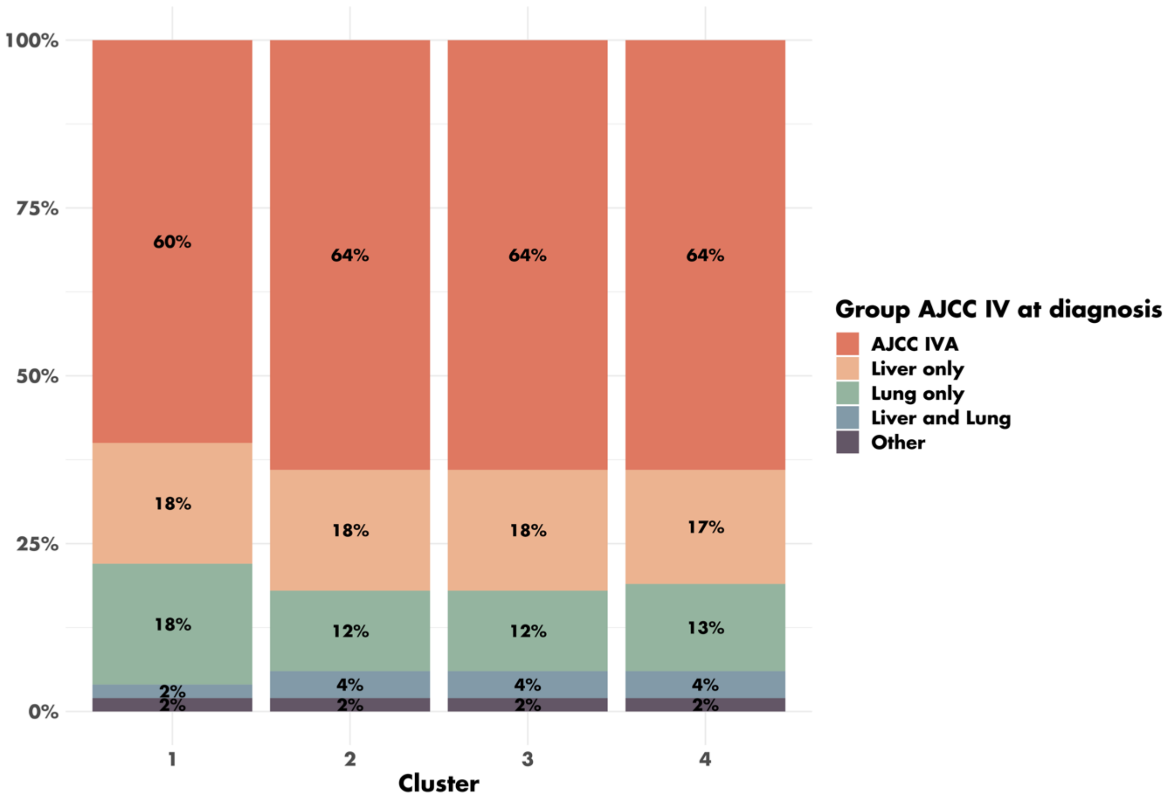

Supplement: Supplementary file 1 [file cancers-14-05987-s001.zip › cancers-2044826-supplementary.pdf]
